# Meaningful Use of Electronic Health Records: Experiences From the Field and Future Opportunities

**DOI:** 10.2196/medinform.4457

**Published:** 2015-09-18

**Authors:** Sarah Patricia Slight, Eta S Berner, William Galanter, Stanley Huff, Bruce L Lambert, Carole Lannon, Christoph U Lehmann, Brian J McCourt, Michael McNamara, Nir Menachemi, Thomas H Payne, S Andrew Spooner, Gordon D Schiff, Tracy Y Wang, Ayse Akincigil, Stephen Crystal, Stephen P Fortmann, Meredith L Vandermeer, David W Bates

**Affiliations:** ^1^ Division of Pharmacy School of Medicine Pharmacy and Health Durham University Durham United Kingdom; ^2^ The Centre for Patient Safety Research and Practice Division of General Internal Medicine Brigham and Womens Hospital Boston, MA United States; ^3^ Center for Health Informatics for Patient Safety/Quality Department of Health Services Administration University of Alabama at Birmingham Birmingham, AL United States; ^4^ University of Illinois at Chicago Chicago, IL United States; ^5^ Department of Biomedical Informatics University of Utah Salt Lake City, UT United States; ^6^ Intermountain Healthcare Salt Lake City, UT United States; ^7^ Center for Communication and Health Department of Communication Studies Northwestern University Chicago, IL United States; ^8^ James M Anderson Center for Health Systems Excellence Cincinnati Children's Hospital Medical Center Cincinnati, OH United States; ^9^ Department of Pediatrics University of Cincinnati Cincinnati, OH United States; ^10^ Pediatrics and Biomedical Informatics Vanderbilt University Nashville, TN United States; ^11^ Duke Clinical Research Institute Duke University Durham, NC United States; ^12^ Departments of Pediatrics and Medical Informatics (DMI) Northwest Permanente Portland, OR United States; ^13^ Richard M Fairbanks School of Public Health Indiana University Indianapolis, IN United States; ^14^ Department of Medicine University of Washington Seattle, WA United States; ^15^ Division of Biomedical Informatics Department of Pediatrics University of Cincinnati College of Medicine Cincinnati, OH United States; ^16^ Harvard Medical School Boston, MA United States; ^17^ Institute for Health, Health Care Policy, and Aging Research Rutgers The State University of New Jersey New Brunswick, NJ United States; ^18^ Center for Health Research Kaiser Permanente Northwest Portland, OR United States

**Keywords:** medical informatics, health policy, electronic health records, meaningful use

## Abstract

**Background:**

With the aim of improving health care processes through health information technology (HIT), the US government has promulgated requirements for “meaningful use” (MU) of electronic health records (EHRs) as a condition for providers receiving financial incentives for the adoption and use of these systems. Considerable uncertainty remains about the impact of these requirements on the effective application of EHR systems.

**Objective:**

The Agency for Healthcare Research and Quality (AHRQ)-sponsored Centers for Education and Research in Therapeutics (CERTs) critically examined the impact of the MU policy relating to the use of medications and jointly developed recommendations to help inform future HIT policy.

**Methods:**

We gathered perspectives from a wide range of stakeholders (N=35) who had experience with MU requirements, including academicians, practitioners, and policy makers from different health care organizations including and beyond the CERTs. Specific issues and recommendations were discussed and agreed on as a group.

**Results:**

Stakeholders’ knowledge and experiences from implementing MU requirements fell into 6 domains: (1) accuracy of medication lists and medication reconciliation, (2) problem list accuracy and the shift in HIT priorities, (3) accuracy of allergy lists and allergy-related standards development, (4) support of safer and effective prescribing for children, (5) considerations for rural communities, and (6) general issues with achieving MU. Standards are needed to better facilitate the exchange of data elements between health care settings. Several organizations felt that their preoccupation with fulfilling MU requirements stifled innovation. Greater emphasis should be placed on local HIT configurations that better address population health care needs.

**Conclusions:**

Although MU has stimulated adoption of EHRs, its effects on quality and safety remain uncertain. Stakeholders felt that MU requirements should be more flexible and recognize that integrated models may achieve information-sharing goals in alternate ways. Future certification rules and requirements should enhance EHR functionalities critical for safer prescribing of medications in children.

## Introduction

The Health Information Technology for Economic and Clinical Health (HITECH) Act was signed into law on February 17, 2009 with the commitment of substantial financial resources to expand the use of electronic health records (EHRs) and great hopes of promoting improvements in the efficiency of health care for all Americans. This effort is being led by the Centers for Medicare & Medicaid Services (CMS) and the Office of the National Coordinator for Health Information Technology (ONC) at the US Department of Health and Human Services (HHS) [1,2]. As a condition for clinicians and hospitals to receive incentive payments, they needed to use certified EHRs in a meaningful manner (ie, “meaningful use” [MU]). More specifically, this involved using EHRs to improve quality, safety, and efficiency; reduce health disparities; engage patients and family in their health; improve care coordination and population and public health; and maintain privacy and security of patient health information [3].

The CMS EHR incentive programs have included 3 stages to date, each with its own specific objectives, measures, and standards. The final rules for MU Stage 1, which specify the criteria that eligible professionals and hospitals need to meet to qualify for incentives, went into effect on September 26, 2010. The rules defined 15 core and 10 menu-set objectives that focused on providers capturing and sharing patient data. The onset of MU Stage 2 criteria was delayed until 2014 and concentrated on advanced clinical processes and more rigorous health information exchange (HIE). Specific to the Stage 2 objectives was the expectation that patients would be provided with secure online access to their health information. The Health Information Technology Policy Committee (HITPC), which advises the government on its EHR incentive program, submitted its preliminary recommendations for MU Stage 3 to the ONC in early 2013. As part of the federal rule-making process, these preliminary Stage 3 recommendations were released for public comment and generated a high volume of responses [4]. These responses play a key role in informing the future direction of MU and related health information technology (HIT) policies, with Stage 2 now extended through to 2016 and Stage 3 scheduled to begin in 2017. At this time, relatively little has been published about professionals’ experiences with implementing the Stage 2 requirements. A large number of these core measures are associated with the entering, recording, or ordering of medicines. Our goals were to examine critically the impact of MU to date, both experiences with Stage 2 and reactions to Stage 3 recommendations, with a particular focus on medication requirements along with related broader policy and implementation issues. We used this information to develop a set of recommendations to help inform future policies.

## Methods

We gathered the perspectives of a wide range of professionals (N=35) representing academicians, practitioners, policy makers, and senior management officials identified through the CERTs, henceforth referred to as “stakeholders.” Stakeholders initially met in June 2014 as part of the national CERT steering committee meeting to discuss the purpose and content of this document and included representatives from different health care and academic organizations including: Agency for Healthcare Research and Quality (AHRQ) (n=5), Kaiser Permanente (n=4), Brigham and Women’s Hospital (n=3), Cincinnati Children’s Hospital Medical Center (n=2), Food and Drug Administration (FDA) (n=2), Duke University (n=3), Rutgers University (n=2), University of Alabama at Birmingham (n=2), Intermountain Healthcare (n=1), University of Illinois at Chicago (n=1), Northwestern University (n=1), University of Washington (n=1), University of Maryland (n=1), Baylor Scott and White Health (n=1), Baylor College of Medicine (n=1), Blue Cross Blue Shield Association (n=1), and a variety of others (n=4). A number of stakeholders occupied roles such as Chief Medical Information Officer or Chief Medical Informatics Officer in their respective health care organizations. A number of open-ended questions were posed to the group including:

- What were your experiences of implementing Stage 2 MU requirements?
- What key challenges did you face?
- How were these challenges overcome (or could they be overcome in the future)?
- What are your thoughts on the proposed Stage 3 recommendations?
- Do you think there were any important areas omitted in the proposed Stage 3 recommendations?

Specific issues and recommendations were presented, discussed, and agreed on as a group. Some of these issues that were agreed on by the stakeholders have been documented and supported by relevant literature. We used the principles of consensus decision making; all stakeholders were (1) involved in the group discussions (inclusive), (2) encouraged to contribute opinions and suggestions (participatory), (3) given the opportunity to build on one another’s suggestions (collaborative), (4) afforded equal input into the process (egalitarian), and (5) allowed to voice any particular concerns that they may have so that the group could incorporate them into the emerging domains (cooperative). These include, for example, how organizational differences in the delivery of health care could impact stakeholders’ ability to achieve MU requirements, challenges and opportunities for rural communities, and how EHRs could be improved to support safer and more effective prescribing for children. The public commentary available on the government website was reviewed to help inform these discussions [4]. A summary of the key findings were presented to the group as an oral presentation (via a webinar) in January 2015 and a concerted attempt was made to reach full agreement on the key domains (principle of agreement seeking). All stakeholders had the opportunity to provide feedback both verbally and electronically, and all feedback was incorporated. The stakeholders were convened for a second face-to-face meeting at the start of March 2015 and gave their final approval to the manuscript’s content and recommendations. All authors listed on this manuscript participated in these meetings. In the sections that follow, we discuss these 6 domains, which include some of the key objective(s) on which the HITPC requested comment and the HITPC identification number to facilitate cross-referencing.

## Results

### Accurate Medication Lists and Medication Reconciliation

When a patient is transferred from one health care setting or provider to another, it is essential that accurate and up-to-date information about the patient’s medications be provided. This enables health care professionals responsible for the patient’s care to identify any medication changes or discrepancies between the prior and current medication lists. This process of medication reconciliation helps health care providers make informed decisions and safely monitor their patients’ care [5]. A Stage 2 core measure recommended that medication reconciliation be performed for more than 50% of patients transitioning into the care of the eligible provider or admitted to the eligible hospital’s or Critical Access Hospital’s (CAHs) inpatient or emergency department (SGRP 302). However, the consensus of the stakeholder group was that this process of medicine reconciliation is very important and requires attention. The quality and accuracy of these medication lists are often poor and providing patients with medication lists that are of dubious quality (due to missing, duplicated, or inaccurate prescription information) can pose a risk to patient safety. Medication lists can also fall short, for example, by excluding important information critical to pediatric dosing, such as the intended weight-based dose, adjustments made based on gestational age, and dose rounding. As part of the medication reconciliation process, prescribers and nonprescribers (eg, medical assistants) are now entering medical information about patient medications such as a report that the patient is not taking a drug. This “not taking” data element fails to capture whether the drug has or has not been prescribed or discontinued, or whether the patient is choosing not to take the medication. The ambiguity in the meaning of the data element “not taking” introduces considerable variation in how individuals handle this information in the EHR system and raises questions on how the quality of this process would be measured or monitored.

Better electronic tools are needed to assist with this medication reconciliation process [6]. For example, 3 stakeholders highlighted how Partners Healthcare developed an electronic postdischarge tool that presents the ambulatory EHR medication list (preadmission) alongside the discharge medication list on the same screen with all differences in dose or frequency highlighted [7]. Medications can then be efficiently added to, updated, or deleted from the EHR medication list. The primary care provider could also “verify” that a medication was up-to-date, thus helping other clinicians judge the accuracy of medication information. This electronic tool is one example of automated approaches that could more actively involve the primary care provider and improve patient safety at the transition from hospital to primary care.

A Stage 3 recommendation was that EHR systems should provide functionality to help maintain an up-to-date accurate medication list (SGRP 106); therefore, the incorporation of external data, such as pharmacy dispense status notifications, into vendor EHR systems was proposed for a future stage of MU (SGRP 125). These data could better inform users as to whether a patient had their prescription(s) filled, was taking 2 kinds of the same drug (including detection of abuse), or was using multiple drugs whose indications overlap. All stakeholders agreed that such needed interoperability poses additional challenges related to data validity, reliability, and integrity, and concerns about the willingness, timing, and ability of pharmacies to make these data available electronically.

One specific recommendation from the stakeholder group was that medication cancelations should be transmitted to pharmacies. This is often done in the inpatient setting, but it is not done in the outpatient setting, although a standard does exist. If this were done, it could help resolve many discrepancies in medication reconciliation.

### Accurate Problem Lists and the Shift in Health Information Technology Priorities

An accurate list of a patient’s problems and allergies represents a key component of the patient’s EHR. Problem lists contain a list of patients’ problems or diagnoses and may be used by clinicians to familiarize themselves with the needs of a patient and orient caregivers to the reasons why a patient may be on a particular medication or regimen. If a problem is properly documented in a patient’s EHR, their clinician can receive appropriate alerts and reminders to guide care. The problem list also helps primary care practices to correctly identify disease-specific populations and create patient registries ensuring that all patients benefit from the most up-to-date evidence-based care.

The MU Stage 3 recommendations expanded the scope of reconciliations to include those of medication allergies and problems (SGRP 302). Many stakeholders recognized the importance of obtaining patients’ input on the accuracy of problem lists (SGRP 105) in the process of reconciliation. However, concerns were raised by the stakeholder group about whether and how patients should contribute to the same up-to-date problem list as clinicians and, if so, whether this may confuse and possibly interfere with the credibility of the list [4]. Some patients do not actually have any active problems and stakeholders grappled with the need to distinguish the explicit absence of a problem from the situation in which a problem may exist but was not entered (or does not fit the criteria that CMS has determined for what constitutes a problem). For example, one stakeholder highlighted how Intermountain Healthcare had asked their physicians to enter problems or “no problems” in the chart to comply with MU, but in actual use, many items on the problem list were not “problems” according to CMS rules and so “no CMS problems” was entered instead. This proved confusing for clinicians to interpret.

One Stage 2 core measure recommended maintaining an up-to-date problem list of current and active diagnoses (SGRP 105) and a medication allergy list (SGRP 107). Stage 3 recommendations expand on these basic requirements proposing that EHR systems should also provide functionality to help keep both problem and allergy lists accurate and up-to-date. One stakeholder explained how the University of Washington has developed new functionality using natural language processing to help achieve this objective for EHR problem lists. However, because of the burden of complying with MU requirements, other work that was not directly tied to MU incentives was postponed or halted. For example, before the launch of the MU incentive program, there were active clinical decision support (CDS) initiatives on-going at the University of Washington for the early detection of sepsis, identifying non-ICU patients at risk of clinical deterioration, complying with guidelines to reduce ventilator-associated pneumonia, venous thromboembolism, and other complications of ICU care—all leading causes of patient harm. However, to meet MU requirements, work on these projects was deferred and the clinical analysts, engineers, and senior programming staff were redirected to work on implementing MU requirements. One stakeholder reported a similar stifling of innovation at Intermountain Healthcare, where the implementation of MU capabilities delayed other EHR development projects, such as the replacement of legacy system functionality in labor and delivery, electronic consent handling, clinical HIE workflow integration, and replacement/enhancement of inpatient computerized provider order entry (CPOE) functionality. The consensus of the stakeholder group was that this might represent an opportunity cost for innovation. Institutions understandably may place priority on innovations that will bring known rewards, even if the innovations would not be as high a priority if there were no incentives. These unintended consequences of the MU incentives can be instructive to consider as other “pay for performance” programs are initiated.

The definition of CPOE by CMS is “a provider’s use of computer assistance to directly enter medical orders (eg, medications) from a computer or mobile device” [8]. The Stage 3 MU measure recommended 60% of medication orders and 60% of laboratory and radiology orders (as opposed to 30% in Stage 2 MU) be recorded by the eligible or authorized provider using CPOE. Stakeholders supported the inclusion of drug-drug interaction (DDI) checking in CPOE systems for “never” combinations (SGRP 101)—combinations that have the potential for severe adverse effects if prescribed together. Questions frequently arose about who would create and maintain such an externally vetted list of DDI alerts for “never” combinations. Two stakeholders suggested that the creation of a national knowledge base, which is managed centrally, might be one possible option to consider so that each organization does not have to individually reinvent the wheel. However, most stakeholders felt the overall utility of DDI alerts was mixed because of a plethora of what clinicians perceived were “nuisance alerts” that they mostly ignored. All alerts need to be implemented thoughtfully with careful attention paid to the balance between sensitivity and specificity, how the alerts are delivered to providers, how intrusive they are to provider workflow, and the provider’s clinical specialty and patient population. Stakeholders felt that organizations should be allowed flexibility in managing DDI alert implementation to ensure that it does not result in too many false-positive warnings and inaccurate or trivial information, resulting in alert fatigue [4].

### Accurate Allergy Lists and Allergy-Related Standards Development

#### Overview

Stage 2 and 3 recommendations require EHRs to maintain active medication allergy lists (SGRP 107). This includes functionality that codes medication allergies and links them to related drug family and code-related reactions. It known that an allergy that is entered as free text in the EHR is neither interoperable across clinical information systems nor easily usable for CDS applications, such as drug-allergy interaction checking. However, the US government has not yet specified which standard terminologies should be used to structure and encode allergy information. The consensus of the stakeholder group was that defining allergy standards will be essential to facilitate both documentation and the exchange of information between health care settings [4]. One stakeholder highlighted how Goss et al [9] defined a set of desirable characteristics to assess allergy standards and terminologies, and conducted an analysis to examine the content coverage of each existing standard terminology within specific domains. Systemized Nomenclature of Medical Clinical Terms (SNOMED CT) was found to fulfill the greatest number of desirable characteristics, whereas RxNorm provided the most comprehensive coverage for representing drug allergens, followed by Unique Ingredient Identifier (UNII) and SNOMED CT. Unfortunately, no single terminology was found to be, by itself, a complete solution. SNOMED CT was the only terminology to contain concepts to represent “no known allergies.” Failure to document positive findings may result in compliance issues and can potentially jeopardize patient safety [9]. There is a lack of validated outcome measures or service accreditation standards, which would allow improved measurement of the quality of allergy services provided [10]. The stakeholder group agreed that further work is needed to develop a common terminology model, which will reconcile overlapping concepts and terms.

#### Supporting Safer and More Effective Prescribing for Children

Stakeholders, especially those from the Cincinnati CERT that specializes in pediatric medication use, expressed concern about the lack of attention paid to pediatric prescribing in the MU criteria. Although Stage 2 and Stage 3 MU objectives required CPOE systems to be used for 60% of medication orders (SGRP 101), EHR functionalities to assist with the prescribing of medications for children have not been specifically mentioned or recommended. This is despite the fact that prescribing medicines for children is reported in the literature to carry disproportionately higher safety risks and be more error prone compared to prescribing for adults [11]. A child’s continuously changing physiology [12] and limited ability to tolerate errors [13,14] require consideration of gestational age, actual age, weight, length, body surface area, and body mass index when prescribing drugs [15]. With almost one-quarter of the US population being children [16], it stands to reason that EHR functionalities should be developed and widely implemented to promote safer pediatric prescribing.

The American Academy of Pediatrics (AAP), AHRQ, and Health Level 7 (HL7) International, have described desirable functionalities for EHRs in pediatric populations. Major areas include immunization management, growth tracking, medication dosing, data norms, and privacy in special pediatric populations [17]. For safe prescribing, pediatric drug dosages are usually best calculated on the basis of body weight [18,19]. Stakeholders pointed out how it is possible for an EHR system to use this value to suggest doses or indeed request that the weight be updated or entered in the system if absent. EHR systems could also help minimize errors in computing of a volume of liquid for a particular dose and round it to a convenient volume to be administered by a caregiver. Because data norms and values (eg, body measurements and vital signs) change continuously with age, EHRs can also assist with the calculation and flagging of abnormal values. Furthermore, they can generate instructions to the pharmacy to dispense the drug in a particular way [17]. Textbox 1 lists EHR functionalities that stakeholders considered important in prescribing for children.

**Electronic health record functionalities that stakeholders considered important in prescribing for children.**

- Weight-based/body surface-based dose calculations and range checks [14]
- Ability to detect erroneously entered weights [14,20,21]
- Display of patient specific units of measure (eg, grams) along with the data values [22]
- Rounding of medication doses to appropriate decimal precision with special consideration of the low-weight patients [23,24]
- Display of data that influenced the final dose and amount in the prescription, particularly to dispensing pharmacists [25]
- Display of normal pediatric dose ranges and advice when no pediatric references exist [26]
- Use pediatric dose ranges for alerts using patient weight/age with soft-stops for adult dose [27]
- Appropriate alerts for age correction for preterm infants, neonates, and low-weight patients [28]
- Recommendation of optimized dispensing format (liquid, tablet, etc) or concentration for the patient [22,29]
- Adolescent patients require a level of confidential care, especially when prescribing medications for reproductive or mental health issues [30,31]

Stage 3 recommendations propose a new measure that would require health care providers to generate and transmit discharge prescriptions electronically (SGRP 103). Although this objective may improve workflow for pediatric providers and reduce the risk of illegible handwriting and transcription errors, the stakeholder group felt that it does not focus on the decision support required to generate correct prescriptions and may simply enable faster generation and transmission of potentially erroneous orders. Current formats for electronic prescription messages do not include body weight or any details about the calculations that yielded the dose [32]. Thus, the consensus of the stakeholder group was that few of the Stage 2 requirements were aligned sufficiently with the functionalities considered critical for the accurate prescribing of medications in children and it was key that this issue be addressed in the development of future recommendations.

#### Challenges and Opportunities for Rural Communities

Awards totaling US $10 million were collectively granted to 5 domestic institutions to support HIT curriculum development in April 2010 to the University of Alabama at Birmingham, Johns Hopkins University, Columbia University, Duke University, and Oregon Health and Science University. Each of these Curriculum Development Centers was given responsibility to develop, revise, and share curriculum components covering a specific set of HIT content areas. The ultimate aim was to prepare future professionals to meet emerging workforce needs. Despite the initial HITECH funding for training, stakeholders felt that the needs of the HIT workforce in rural areas across the country have not been met yet. Rural communities are more likely to have smaller practices, which have been among the last to embrace electronic medical records [33]. They have fewer resources to both purchase EHRs and to hire and retain HIT support staff. The overall IT infrastructure in many of these areas (as in some low-resource urban areas) is poor, which makes it even more challenging to participate in the electronic information exchange. Thus, patients with complex conditions in rural communities may not benefit from the quality improvements that the MU incentives are designed to deliver.

According to the stakeholders, especially those from the University of Alabama at Birmingham CERT that specializes in workforce training, several steps have been taken to address these issues. In addition to the workforce training programs, 62 Regional Extension Centers (RECs) have been established with US $677 million in funding from the ONC to provide on-the-ground assistance to smaller rural practices. In 2011, the ONC announced an additional US $12 million in new technical support assistance to help CAHs and rural hospitals adopt and become meaningful users of certified HIT. This funding was in addition to the $20 million provided to RECs in September 2010 to provide technical assistance to the CAHs and rural hospitals [34]. In addition, University of Alabama at Birmingham and Columbia University collaborated with representatives from several of the other RECs to adapt the original training curriculum so that it would be better suited to the needs of rural and low-resource urban practices. In 2013, the Health Services and Resource Administration (HRSA) funded rural networks in 15 states to develop rural HIT workforce development programs to provide education, apprenticeships, and job placements in rural practices [35]. HRSA, AHRQ, and ONC have also developed resources, checklists, and toolkits to help sites unable to afford expensive outside consultation [36].

One stakeholder pointed out that as more hospitals and practices begin to meet the MU criteria, some of the traditional boundaries that have separated rural primary care practices from tertiary care centers in large urban areas may begin to disappear. Primary care practices may have more access to information about their patients’ hospitals stays. Tertiary care hospitals are likely to have a substantial number of patients from surrounding rural areas who can benefit from patient portals or similar mechanisms to promote patient engagement (SGRP 204A). However, patient engagement is likely to be another challenge going forward with rural residents, considering unreliable Internet connections, low health literacy, and lack of resources. Although MU requirements currently set a low percentage of patients who are expected to use the portals, the consensus of the stakeholder group was that systems must be scalable if more patients are to benefit, which will likely entail use of novel technologies such as mobile devices.

#### Achieving Meaningful Use: Easier for Some Than for Others?

Many different stakeholders supported the MU general goal that providers should have appropriate information about patients transitioning into their care (SGRP 303). Stage 3 recommendations expanded on this Stage 2 objective by specifying the types of information that should be included in the summary care record, such as a concise narrative section, goals, instructions, and care team members. The consensus of the stakeholder group was that some organizations, such as Kaiser Permanente or Intermountain Healthcare, might find it easier to achieve this objective than others. Such well-established integrated delivery systems have organized, coordinated, and collaborative networks that bring together various health care providers to deliver coordinated care to a defined patient population [37]. They include primary and specialty outpatient care, as well as community and tertiary hospital services. The effective use of HIT is a key attribute of successful integrated delivery systems [37,38]. For example, in the case of Kaiser Permanente or the Veterans Affairs systems, the same longitudinal EHR is accessible and shared by both primary care physicians and specialists, thus facilitating the tracking of patients across the continuum [38]. Kaiser Permanente also has an integrated pharmacy system that is used for most patient prescriptions. One stakeholder highlighted how, for the past 20 years, Kaiser Permanente has had a bidirectional electronic HL7-based interface in place in their pharmacy systems, which has ensured that the information presented to their patients was consistent, whether they were engaged with clinical operations, outpatient pharmacy locations, or mail order pharmacy services. It also meant that the Stage 2 recommendation to generate and transmit permissible discharge prescriptions electronically (SGRP 103) was easily achievable for all eligible providers. However, this stakeholder also explained how other measures, such as Summary of Care documentation at time of transitions with external organizations, have required substantial resources to fund technical and operational change that has impacted less than 2% of Kaiser Permanente’s patient population. Care should be taken to avoid MU requirements that are unnecessarily burdensome to mature, typically staff model systems that have historically been the leaders in integrated use of clinical information.

Another issue raised by a different stakeholder related to whether organizations are using existing functionality (eg, Surescripts) or have chosen to develop their own. Kaiser Permanente and other integrated delivery systems lacked the functionality to bring medication information from external pharmacies into their EHR system and were swayed by the MU incentives to add this to their systems. However, the value of this functionality within staff model systems such as Kaiser Permanente is likely to be low in light of the fact that Kaiser Permanente patients obtain nearly all their medications from Kaiser Permanente. Stakeholders agreed in principle that external interoperability functionality can help maintain accurate medication and problem lists, although they felt that implementation should be flexibly based on the organizational-specific contexts. They also felt that many of the specific criteria should be postponed until the technological, operational, and legal issues are more fully evolved and the quality and accuracy of tools are sufficiently tested.

Finally, Stage 3 recommendations propose a new measure that would require health care providers to use CPOE for referrals/transition of care orders (SGRP 130). One stakeholder highlighted how some organizations, including Intermountain Healthcare, already use extensive CPOE/CDS capabilities and other advanced functionality and questioned the value of spending considerable resources to develop functionality that they believed would add little to their existing systems simply to meet MU requirements. For example, for the successful attestation of Stage 1, Intermountain Healthcare estimated that its 696 eligible professionals and 22 hospitals were eligible for approximately US $46.3 million. The high degree of coordination already inherent in their delivery model and IT systems meant that total costs for the implementation of Stage 1 recommendations were considerably lower than for others at an estimated US $17.3 million, resulting in a net revenue benefit of US $29 million. Although this financial benefit may seem substantial, another stakeholder pointed out how these total implementation costs may not reflect the “true” cost because they did not include the development of the system’s computer network (in their case, this was already in existence) or the disruption caused by HIT implementations and upgrades. Therefore, the consensus of the stakeholder group was that it is important to understand the current structural advantages of existing integrated delivery systems in the achievement of MU objectives and to recognize the need for future MU requirements to be applied and interpreted more flexibly. Textbox 2 lists a summary of the key issues for each domain.

**A summary of the key issues in each domain.**

1. Accurate Medication Lists and Medication Reconciliation

- The quality and accuracy of these medication lists is often poor and providing patients with medication lists that are of dubious quality can pose a risk to patient safety.
- Better electronic tools are needed to assist with this medication reconciliation process.
- The incorporation of external data, such as pharmacy dispense status notifications, into vendor EHR systems could better inform providers about a patient’s medicines usage.

2. Accurate Problem Lists and the Shift in HIT Priorities

- EHR systems should also provide functionality to help keep both problem and allergy lists accurate and up-to-date.
- Institutions understandably may place priority on innovations that will bring known rewards, even if the innovations would not be as high a priority if there were no incentives.
- All CDS alerts need to be implemented thoughtfully with careful attention paid to the balance between sensitivity and specificity, and how the alerts are delivered to providers.

3. Accurate Allergy Lists and Allergy-Related Standards Development

- Defining allergy standards will be essential to facilitate both documentation and the exchange of information between health care settings.

4. Supporting Safer and More Effective Prescribing for Children

- Data norms and values change continuously with age and EHRs can assist with the calculation and flagging of abnormal values.
- Few Stage 2 requirements were aligned sufficiently with the functionalities considered critical for the accurate prescribing of medications in children and it was key that this issue be addressed in the development of future recommendations.

5. Challenges and Opportunities for Rural Communities

- Despite the initial HITECH funding for training, the needs of the HIT workforce in rural areas across the country have not been met.
- Patient engagement is likely to be challenge going forward with rural residents, considering unreliable Internet connections, low health literacy, and lack of resources.
- Although MU requirements currently set a low percentage of patients who are expected to use the portals, systems must be scalable if more patients are to benefit, which will likely entail use of novel technologies such as mobile devices.

6. Achieving MU: Easier for Some Than for Others?

- Some MU measures have been easily achievable for integrated delivery systems, whereas other measures have required substantial resources to fund and have impacted only a small portion of their patient population.
- Future MU requirements need to be applied and interpreted more flexibly.

## Discussion

We assessed stakeholders’ learning and experiences from the implementation of MU requirements over the past 4 years, with a particular focus on medication requirements and attempted to identify problem areas where midcourse corrections might be helpful. Six specific issues were highlighted, all of which present opportunities for improvement. The implementation of MU capabilities was reported to have stifled innovation at some organizations. This appears to run counter to the ONC’s goal of encouraging innovation and creating “an environment of testing, learning, and improving, thereby fostering breakthroughs that quickly and radically transform health care” [39]. The challenge in many organizations was that resources were largely focused on implementing basic MU criteria and diverted away from addressing other meaningful local problems and creating innovative solutions.

Likewise, although the EHR incentive program was viewed as a valuable opportunity to encourage provider-level clinical quality measure (CQM) innovation and perform provider-level CQM testing, some stakeholders felt it distracted them at least temporarily from their efforts to develop and implement such quality measurement and improvement systems. The HITPC also raised the possibility of allowing health care organizations to submit a locally developed CQM as a menu item, in lieu of one of the existing measures specified in the MU program [3]. Health care organizations may find this difficult to achieve, especially if their clinical analysts, engineers, and senior programming staff are focused on achieving MU requirements rather than other EHR development projects. Furthermore, the Food and Drug Administration Safety and Innovation Act (FDASIA) working group was clear that any new HIT regulatory framework should promote innovation rather than stifle it [40]. This FDASIA working group recommended more local HIT configuration and integration, as well as more control and accountability for outcomes of use. A greater emphasis should be placed on local HIT configuration that addresses population health needs. Thus, MU requirements will need to change and evolve over the next few years to achieve this broader and more flexible orientation. The concerns we have identified have spurred the following recommendations:

1. Definitions of transitions in care should enable and support shared patient record systems. Better tools and interoperability with external data are needed for effective and efficient medication reconciliation. On the other hand, measures should not drive unnecessary or unreliable data transmission.
2. Development of a common terminology model is needed to facilitate documentation and encoding of key data elements, notably patient allergies.
3. Future MU certification rules and requirements should consider EHR functionalities that are critical, but often lacking, for the accurate prescribing of medications in children.
4. Future MU requirements should put more emphasis on flexibly understanding, incorporating, and supporting local HIT configurations that address population health needs.
5. The MU objectives should acknowledge the diversity of health care systems. For example, integrated delivery systems are more likely to achieve the goal of information sharing because of their integrated structure, greater functionality, and improved interoperability. From the policy perspective, this could be handled by offering exceptions or alternate routes for qualification.

The sampling strategy used in this study ensured that the perspectives of highly knowledgeable informants from the 5 AHRQ-sponsored CERTs were captured. Our sample included those directly involved in the implementation of MU criteria (eg, Chief Medical Information Officer or Chief Medical Informatics Officer) and those who were knowledgeable about, but not directly involved in, the day-to-day implementation work (eg, academicians, practitioners, policy makers). Participants were free to raise any issues that they felt were relevant to the topic under discussion. Consequently, we believe that the information gathered was reflective of genuine concerns and views. All stakeholders were given an opportunity to provide feedback on the key domains, ensuring that the conclusions accurately reflected the opinions and views collected. A limitation of this study was that it was performed in the US context and, therefore, could be viewed as less applicable to other countries. However, we believe that the implementation and adoption of EHRs is highly heterogeneous across health care systems and countries, and will be of interest.

The future course that the federal government will take with respect to HIT and policy measures is uncertain. It is not clear whether there will be a fourth stage of MU, although that currently seems unlikely. Taking stock of the important ways MU has been successful in achieving many of its objectives—such as dramatically increasing the number of medications ordered electronically—as well as where it encountered predicted and unanticipated problems, will be critical to mapping the next steps. Overall, the incentives and specific MU criteria will almost certainly be less important than they have been in the future as information systems more broadly improve their functionality and many of the challenges that we face today become embedded as the standard of care. It does appear that certification will continue to be important, although providers have recently called for separating MU from certification [41]. The ONC will likely continue to (appropriately) maintain its “bully pulpit” role in helping to encourage and accelerate the development of standards and interoperability among other needs. Finally, it appears likely that a national Center for HIT Safety will be established, a development many of the CERT stakeholders welcomed, especially given the valuable role CERTs have historically played in the coordination of national medication improvement efforts [39].

Regardless, we believe it will be important for the federal government to address some of the issues we have identified in this paper, including problems with how medication reconciliation is being promoted, the issues around accurate problem lists and the shift in HIT priorities, supporting safer and effective prescribing for children and rural communities, and making achieving MU more likely to result in the care improvement desired by all stakeholders. Any new policy will introduce new problems and it is essential for the federal government and others to consider how best to address these issues and others through the MU incentive program.

## Abbreviations

CAH: Critical Access Hospital
CDS: clinical decision support
CERT: Centers for Education and Research in Therapeutics
CPOE: computerized provider order entry
CQM: clinical quality measure
DDI: drug-drug interaction
EHR: electronic health records
FDASIA: Food and Drug Administration Safety and Innovation Act
HIE: health information exchange
HIT: health information technology
HITECH: Health Information Technology for Economic and Clinical Health
HITPC: Health Information Technology Policy Committee
HRSA: Health Services and Resource Administration
MU: meaningful use
REC: Regional Extension Centers
UNII: Unique Ingredient Identifier
