# Metadata Correction: Meaningful Use of Electronic Health Records: Experiences From the Field and Future Opportunities

**DOI:** 10.2196/medinform.5160

**Published:** 2015-09-25

**Authors:** Sarah Patricia Slight, Eta S Berner, William Galanter, Stanley Huff, Bruce L Lambert, Carole Lannon, Christoph U Lehmann, Brian J McCourt, Michael McNamara, Nir Menachemi, Thomas H Payne, S Andrew Spooner, Gordon D Schiff, Tracy Y Wang, Ayse Akincigil, Stephen Crystal, Stephen P Fortmann, Meredith L Vandermeer, David W Bates

**Affiliations:** ^1^ Division of Pharmacy School of Medicine Pharmacy and Health Durham University Durham United Kingdom; ^2^ The Centre for Patient Safety Research and Practice Division of General Internal Medicine Brigham and Womens Hospital Boston, MA United States; ^3^ Center for Health Informatics for Patient Safety/Quality Department of Health Services Administration University of Alabama at Birmingham Birmingham, AL United States; ^4^ University of Illinois at Chicago Chicago, IL United States; ^5^ Department of Biomedical Informatics University of Utah Salt Lake City, UT United States; ^6^ Intermountain Healthcare Salt Lake City, UT United States; ^7^ Center for Communication and Health Department of Communication Studies Northwestern University Chicago, IL United States; ^8^ James M Anderson Center for Health Systems Excellence Cincinnati Children's Hospital Medical Center Cincinnati, OH United States; ^9^ Department of Pediatrics University of Cincinnati Cincinnati, OH United States; ^10^ Pediatrics and Biomedical Informatics Vanderbilt University Nashville, TN United States; ^11^ Duke Clinical Research Institute Duke University Durham, NC United States; ^12^ Departments of Pediatrics and Medical Informatics (DMI) Northwest Permanente Portland, OR United States; ^13^ Richard M Fairbanks School of Public Health Indiana University Indianapolis, IN United States; ^14^ Department of Medicine University of Washington Seattle, WA United States; ^15^ Division of Biomedical Informatics Department of Pediatrics University of Cincinnati College of Medicine Cincinnati, OH United States; ^16^ Harvard Medical School Boston, MA United States; ^17^ Institute for Health, Health Care Policy, and Aging Research Rutgers The State University of New Jersey New Brunswick, NJ United States; ^18^ Center for Health Research Kaiser Permanente Northwest Portland, OR United States

During article production, the co-author Meredith Vandermeer, MPH (Center for Health Research, Kaiser Permanente Northwest, Portland, OR, United States) was omitted from the list of authors in the paper “Meaningful Use of Electronic Health Records: Experiences From the Field and Future Opportunities” (JMIR Med Inform 2015;3(3):e30). The author Vandermeer should have been added after Stephen P Fortmann in the original published manuscript. This error has been corrected in the online version of the paper on the JMIR Med Inform website on Sept 25, 2015, together with publishing this correction notice. This was done before submission to PubMed Central and other full-text repositories. This correction notice has been submitted to PubMed and the metadata has been resubmitted to CrossRef with publishing this correction notice.

